# Economic and environmental assessments of combined genetics and nutrition optimization strategies to improve the efficiency of sustainable pork production

**DOI:** 10.1093/jas/skab051

**Published:** 2021-02-15

**Authors:** Tara Soleimani, Susanne Hermesch, Hélène Gilbert

**Affiliations:** 1 GenPhySE, Université de Toulouse, INRAE, ENVT, 31326 Castanet-Tolosan, France; 2 Animal Genetics and Breeding Unit, A Joint Venture of NSW Department of Primary Industries and the University of New England, University of New England, Armidale, NSW 2351, Australia

**Keywords:** bio-economic model, environmental assessment, feed efficiency, genetic, pig, residual feed intake

## Abstract

We evaluated the economic and environmental impacts of strategies that incorporated selection for pig feed efficiency and dietary optimization based on a single or multiple objectives tailored to meet the population nutritional requirements, with the goal to optimize sustainable *farm feed efficiency.* The economic and environmental features of the strategy were evaluated using life cycle assessment (**LCA**) and bio-economic models. An individual trait-based LCA model was applied to evaluate global warming potential, terrestrial acidification potential, freshwater eutrophication potential (**EP**), and land occupation of the combined genetics and nutrition optimization to produce 1 kg of live pig weighing 120 kg at the farm gate. A parametric individual trait-based bio-economic model was developed and applied to determine the cost breakdown, revenue, and profit to be gained from a 120-kg live pig at the farm gate. Data from two genetic lines with contrasted levels of feed efficiency were used to apply the combined genetics and nutrition optimization: accounting for the average nutritional requirements for each line, the individual pig responses to diets formulated for least cost, least environmental impacts, or minimum combination of costs and environmental impacts objectives were predicted with INRAPorc. Significant differences in the environmental impacts (*P* < 0.0001) and profit (*P* < 0.05) between lines predicted with the same reference diet showed that selection for feed efficiency (residual feed intake) in pigs improves pig production sustainability. When pig responses were simulated with their line-optimized diets, except for EP, all the line environmental impacts were lower (*P* < 0.05) than with the reference diet. The high correlations of feed conversion ratio with the environmental impacts (> 0.82) and the profit (< −0.88) in both lines underlined the importance of feed efficiency as a lever for the sustainability of pig production systems. Implementing combined genetics and nutrition optimization, the inherent profit and environmental differences between the genetic lines were predicted to be reduced from 23.4% with the reference diet to 7.6% with the diet optimized jointly for economic and environmental objectives (joint diet). Consequently, for increased pig sustainability, diet optimization for sustainability objectives should be applied to cover the specific nutritional requirements arising in the herd from the pigs genetic level.

## Introduction

Improvement in feed efficiency in pigs can be achieved through genetic selection for feed efficiency as feed efficiency itself (gain:feed), feed:gain, its inverse, or residual feed intake (**RFI**) and through diet formulation tailored to the animal’s nutritional requirements and optimized to achieve additional objectives. These approaches, alone or combined, have led to the emergence of different feed efficiency scenarios for better production sustainability, some of which have been the subject of separate investigations. Selection for feed efficiency based on the measurement of RFI and feed conversion ratio (**FCR**, feed:gain) has been successfully implemented in pigs (Gilbert et al., 2007; Cai et al., 2008; Clutter, 2011; Gilbert et al., 2017). The environmental impacts of selection for feed efficiency based on RFI were investigated, for instance by Soleimani and Gilbert (2020). Improving feed efficiency and reducing environmental impacts by feeding animals with diets tailored to their nutritional requirements based on the precision feeding concept have also been investigated (Pomar et al., 2009; Monteiro et al., 2016; Remus et al., 2019), and appropriate methods, decision support tools, and systems are currently under development (Brossard et al., 2017, 2019). Mackenzie et al. (2016), Tallentire et al. (2017), and Garcia-Launay et al. (2018) proposed a variety of diet optimization protocols based on single or multiple objectives. The environmental impacts of feed efficiency improvement scenarios combining genetics, tailored diet formulation, and environmental optimization were investigated by Soleimani and Gilbert (2021). However, a joint evaluation of the economic and environmental impacts of these approaches is still needed to examine how these two pillars of sustainability can best be combined. It will then be possible to perform animal selection and multi-objective diet optimization tailored to the nutritional requirements of each line to improve sustainable farm feed efficiency. The economics of a biological process can be evaluated using bio-economic models (Kragt, 2012), which translate biological components into economic indicators through a system of equations (Dekkers et al., 2004). Bio-economic models can be based on either a deterministic approach, in which mean values are input parameters (Brascamp, 1978), a stochastic approach, in which the mean and variances of the input parameters are used (Jones et al., 2004), or a combination of stochastic and deterministic approaches (Michaličková et al., 2016). For environmental assessment, life cycle assessment (**LCA**) has become the standard framework to assess the different aspects of pig production systems (Lammers, 2011; McAuliffe et al., 2016, 2017). In this study, a trait-based bio-economic model was designed and developed to simulate the profit to be made from each individual pig directly using its own traits. When applied to a set of different individuals, it enabled the estimation of the variability of the profit at the farm level. This model was used jointly with our previously developed LCA model, which incorporates the individual performance traits of fattening pigs, to perform the LCA of individual pigs (Soleimani and Gilbert, 2020). The aim of the present study was thus to evaluate the sustainability of several combined genetics and nutrition optimization scenarios in terms of economy and environment, using individual deterministic bio-economic and LCA models to quantify the economic and environmental costs of different optimization options combining diets and pig genetics. Performing individual assessments also provides insights into the correlations between production traits, profit, and environmental impacts, which can then be used for further optimization of selection and management of pig production systems.

## Material and Methods

### Animal data

All procedures involving animal data collection were in accordance with the national regulations for humane care and use of animals in research. This section provides an overview of the origin of the experimental data, collection procedures, and tools and of the application to set up the growth performance profile of the individual pigs. A scheme of the procedure implemented for economic and environmental assessment of combined genetics and nutrition optimization scenarios is presented in Supplementary Material 1.

#### Experimental data

Experimental data were collected from birth to slaughter from the fifth generation of Large White pigs divergently selected for RFI (Gilbert et al., 2017) in the experimental facilities at INRAE (Surgères, France, https://doi.org/10.15454/1.5572415481185847E12). RFI is defined as the difference between observed feed intake (**FI**) and FI predicted from maintenance and production requirements. The present dataset included 57 male pigs from each of the low RFI (**LRFI**, more efficient pigs) and high RFI (**HRFI**, less efficient pigs) lines. Fattening pigs had ad libitum access to a one-phase conventional diet. The daily FI of each individual was recorded by ACEMA 64 automatic feeders (ACEMO, Pontivy, France) from 11 wk of age to 110 kg live weight (**LW**). Body weight was recorded at birth, at weaning (at average 28 d of age), at the beginning of the growing period (10 wk of age), and at least once a month during fattening until slaughter (average body weight [**BW**] at slaughter: 110 kg), and average daily gain (**ADG**) and average daily feed intake (**ADFI**) for the fattening period were computed. Back fat thickness (**BFT**) was measured using an ALOKA SSD-500 echograph on live animals at 23 wk of age (Aloka, Cergy Pontoise, France). The selection procedure and results are reviewed in Gilbert et al. (2017) for both LRFI and HRFI lines.

#### Growth model and individual profiles

The recorded experimental data for all fattening pigs were imported into the population version of InraPorc (Brossard et al., 2014), which simulates the performance of pigs in response to different nutritional strategies (van Milgen et al., 2008). The imported data were first used to calibrate an individual growth performance profile based on the Gompertz growth function for each pig. The profiles for the fattening period were calibrated according to the daily ad libitum NE uptake using the Gamma function. The calibrated profiles were then used to estimate the FI of pigs when offered different optimized diets to simulate the individual performance responses of pigs up to slaughter weight. A fixed LW of 120 kg at slaughter was applied to facilitate the comparison of the economic and environmental outcomes of the different scenarios. The resulting traits and animal indicators (ADFI, ADG, BFT, lean meat percentage [**LMP**], carcass weight, age at slaughter, and fattening duration) for each individual were used as input parameters for economic and environmental assessment with the bio-economic and LCA models described in the following section.

### Bio-economic model

#### General structure

The bio-economic model was developed in R using a typical linear profit model (Janssen and van Ittersum, 2007). The linear profit model calculates profit as sales revenue minus costs. In this model, the life cycle of a market pig is assumed to be divided into three periods: up to weaning (~28 d of age), postweaning (~28 to 75 d of age), and growing-finishing (~ 75 d of age to reach 120 kg BW):

$$\begin{array}{ll} Costs\;\left(120\;kg\;live\;pig\right)\;= & \;weaned\;piglet\;market\;price\;\\ & +\;postweaning\;costs\;\\ & +\;growing\text{-}f\;inishing\;costs \end{array}$$

All costs related to reproduction (sow plus litter), including artificial insemination and replacement costs, health costs, energy, feed, maintenance, labor force, manure disposal, and capital depreciation, were included in the market price of a weaned piglet. Since LRFI sows produced more weaned piglets than HRFI sows (10.2 LRFI vs. 9.6 HRFI; Gilbert et al., 2012) and the lactation FI of LRFI sows was lower than that of HRFI sows (4.54 kg/d for LRFI vs. 4.82 kg/d for HRFI; Gilbert et al., 2012), using the same weaning costs for the two lines resulted in a conservative hypothesis for LRFI pigs. Postweaning costs were calculated using the experimental data collected from the beginning to the end of postweaning in the two lines. The required data including ADFI, ADG, diet types, and feeding duration are reported in Gilbert et al. (2019). The fattening costs were calculated based on individual traits. The revenue from each pig was only that obtained from the sale of live pigs at the farm gate, which is equal to the market price of the pig. The cost of manure treatment and application from weaning to finishing was assumed to be offset by its revenue. The values and market prices of the services and raw materials were taken from French and European references. The output of the model is the profit made on an individual 120-kg live pig at the farm gate.

#### Breakdown of costs

The costs of fattening including feed and water, building and capital, and energy and labor costs were parametrized individually with performance traits. Other costs including insurance, veterinary care, health, maintenance, and repairs were considered as fixed costs. The cost of each component is summarized in Supplementary Material 2.

##### Feed and water costs.

Feed and water costs were assumed to be the cost of uptaken feed and water. The cost of feed after weaning was calculated based on a conventional two feed phase dietary sequence, with a starter diet from weaning to day 12 and a postweaning diet until the end of the postweaning period. The ADFI (kg/d) of the two diets in each line under ad libitum access to feed is reported in Gilbert et al. (2019). The cost of feed was calculated by multiplying the average quantity of feed consumed at each stage by the price of the feed in France. During fattening, the cost of feed for each individual pig was obtained by multiplying the price of a 1 kg fattening diet (€/kg) by ADFI (kg/d) and the duration of the fattening period (d) of the pig concerned. The price of each ingredient was calculated from the monthly average market price of the ingredients in France reported in the monthly information pamphlet on feed published by the pig industry (IFIP – *Institut de la Filière Porcine*, *Mensuel d’information aliment*, May 2020). The cost of drinking water was considered to be proportional to feed consumption, multiplied by the price of drinking water (€/liter). The water to feed ratio was considered to be 2.5 liters/kg of feed in the postweaning stage (IFIP, 2014). The water to feed ratio was 2.7 liters/kg of feed during the fattening period (IFIP, 2014). The price of water was obtained from the water industry’s information center in France (https://www.cieau.com/le-metier-de-leau/prix-des-services-deau/).

##### Cost of energy.

The cost of energy during the postweaning period in each line was calculated by multiplying the individual ADG and the duration of the postweaning stage by the energy consumption per kilogram of weight gain (0.42 kWh/kg of gain; IFIP, 2014) and the cost of energy (€/kWh) in France. The cost of energy during the fattening period was calculated by multiplying individual ADG and fattening duration (d) by 0.42 (kWh/kg of gain) by the price of energy (€/kWh) in France.

##### Cost of labor.

The cost of labor was calculated based on the French reference, which is of 2.3 farm workers for a farm with 200 sows, with 25 weaned piglets per sow per year, 1,600 working hours per year, and the cost per hour of a labor earning the minimum wage (1.5 * min. wage/h; min. wage = 10.03 €/h). The cost of labor was broken down into the cost of labor per pig and per day (€/pig/d) and then multiplied by the duration of the postweaning and fattening to compute the cost of labor for an individual pig at the farm gate.

##### Buildings and capital costs.

Buildings and capital costs were calculated as the investment required per sow, assuming 25 weaned piglets per sow per year on average and an interest rate of 6% per year. Annual depreciation was included in the sales price of a weaned piglet. The capital cost for an individual pig was estimated by multiplying the capital cost per pig and per day (€/pig/d) by the duration of the postweaning and fattening periods.

#### Revenue

Revenues are represented by the finishing pig market price. The revenue from selling the cull sows was assumed to be included in the market price of a weaned piglet. In the French market pricing system, the price of a finishing pig is a multivariate function of quantity (carcass weight), quality of the carcass (LMP), and a bonus or penalty per kilogram carcass depending on the combined values of these two parameters (Supplementary Material 3; Lopez et al., 2016). The individual market prices were estimated based on the pig carcass traits simulated by InraPorc for each diet. The base market price of the carcass was calculated using the market price of a 100-kg carcass and LMP of 56% (https://rnm.franceagrimer.fr/prix?PORC).

#### Profit

The profit per pig (€/pig) was obtained by subtracting the individual production costs from the revenue obtained by the sale of the finished pig. The formulations were used to calculate the individual profit (see Supplementary Material 4).

### Environmental assessment

#### LCA choices

A “cradle-to-farm-gate” system boundary was built using typical French pig farming systems, including sow litter, postweaning, fattening pigs, feed production, and manure management, which is schematically depicted in Soleimani and Gilbert (2020). One kilogram LW of pig at the farm gate was chosen as the functional unit to enable reliable comparison of the environmental impacts of the different assessments. The impact categories that contributed most to emissions during housing of the animals, manure storage, and application (de Vries and de Boer, 2010) were selected for analyses first: global warming potential (**GWP**, kg CO_2_ eq), acidification potential (**AP**, kg SO_2_ eq), and eutrophication potential (**EP**, kg P eq), which are also the most conventional impact categories in LCA of pig production systems (McAuliffe et al., 2016). Moreover, in pig farming, feed production accounts for almost 100% of the land occupation (**LO**, m^2^a crop eq) impact category (Basset-Mens and van der Werf, 2005) and thus was included in our analysis. The method of ReCiPe Midpoint 2016 (H) V1.02 (Huijbregts et al., 2017), the Ecoinvent inventory (Wernet et al., 2016), and Ecoalim (Wilfart et al., 2016) databases were used to assess environmental impacts. Based on the same approach as in a previous study using this model (Soleimani and Gilbert, 2020), the individual environmental impacts of each pig in the two lines were assessed on the MEANS (MulticritEria AssessmeNt of Sustainability) platform using SimaPro V8.5.4.0 (http://www.inra.fr/means).

#### The LCA model

Briefly, the LCA model was developed in six modules based on net energy: animal profile, feeding plan, emissions, excretion, water expenditure, and energy expenditure (Soleimani and Gilbert, 2020). In addition to the R and InraPorc module to decipher individual profiles during the postweaning and fattening stages described previously, we also used the sow version of the InraPorc software (Dourmad et al., 2008) to set up a single sow-litter profile per line for all assessments. Energy and water expenditure were calculated based on a report on typical French farms by the IFIP (2014). For individual LCAs, the fattening performance traits of each pig were used as input parameters in the life cycle inventory in SimaPro. Using the mass balance approach, the composition of the excreta (dry matter [DM], organic matter [OM], potassium, phosphorus, and nitrogen) was calculated as the difference between nutrient intake and the nutrients retained in the body (Supplementary Material 5). Individual performance data were used for the postweaning and fattening stages, and average performance data were used for the sow-litter stage. The building emissions of ammonia, nitrogen monoxide, enteric methane, nitrous oxide, and nitrogen were calculated following Rigolot et al. (2010a, 2010b). The guidelines provided by the intergovernmental panel on climate change (IPCC, 2006) were used to calculate emissions of methane, direct and indirect emissions of nitrous oxide, and leaching of phosphate and nitrate during the spreading of slurry. Emissions of ammonia during outside storage were calculated based on the emission factors recommended by Rigolot et al. (2010b). Emissions of nitrogen oxides were calculated following Nemecek et al. (2004). As a replacement for synthetic fertilizer, the fertilizer equivalence value of the manure was considered to be 75% for nitrogen (Nguyen et al., 2010) and 100% for phosphorus and potassium (Nguyen et al., 2011). To be sure that the results were consistent and comparable, the same inventories, methods, and calculations were used in all the LCA runs. Using the Ecoalim dataset (Wilfart et al., 2016) of the AGRIBALYSE database, the environmental impacts of the diet ingredients were estimated by applying the ReCiPe Method (Huijbregts et al., 2017). A distance of 100 km was assumed for the transport of the ingredients of the diets from the farm to the feed factory, a distance of 500 km for cereals (Garcia-Launay et al., 2018), and a distance of 30 km (Cadero et al., 2018) for transport from the feed factory to the pig farm, using the Ecoinvent version 3.1 database (attributional life cycle inventories).

### Diet optimization

#### Choice of ingredients

Six new ingredients (corn, oats, peas, triticale, rapeseed meal, and sunflower meal) were added to the eight ingredients of the reference commercial diet (wheat, barley, soybean meal, sunflower oil, and synthetic l_lysine, l_threonine, l_tryptophan, and dl_methionine), giving a total of Q = 14 ingredients incorporated in the diet formulation. The reference diet was a commercial French conventional experimental diet offered to the animals during the experimental data collection (as fed in 2005). It was thus formulated to allow the expression of the genetic potential of all pigs, with a low-cost constraint. The new ingredients were chosen to extend the choice of protein and energy resources based on the availability of data on their impacts, their cost, and their market availability. Information concerning digestible crude protein (CP), amino acids (AA), and net energy (NE) density of the ingredients was obtained from the feed ingredients database INRA-AFZ (Sauvant et al., 2004). Considered as additives, ingredients that have no digestible CP or AAs or energy (e.g., salt, calcium carbonate, and vitamins) were not included in diet formulation. However, their nutritional properties and the potential nutritional shortcomings that could arise from their inclusion in the optimized diets would be picked up by the InraPorc software in the simulations of the individual responses to these diets. Some commercial and industrial limitations for diet optimization, like the possible incompatibility of the list of ingredients to feed milling and processing constraints, were not accounted for in this study either but, in practice, may represent notable constraints.

#### Definition of the nutritional requirements of each line

To be able to identify the nutritional constraints to tailored diet formulation, the dietary requirements of the species concerned have to be known. Pigs adjust their ad libitum FI to the dietary NE density (Quiniou and Noblet, 2012) so that the nutrients in the diet are taken up in proportion to the NE of the diet. In addition, balanced nutritional composition relies on certain essential AAs, such as lysine, threonine, tryptophan, and methionine, which are usually added to cereals as they are most limiting AAs in cereal-based diets (D′Mello, 1993). To avoid AA deficiency, the four abovementioned amino acids were considered as constraints in the formulation of the diets tested in the present study. To ensure the remaining essential and nonessential amino acids were covered, the requirements for digestible CP per MJ NE were also obtained for each individual from InraPorc and considered among the constraints. Finally, to account for the fact that FI is regulated by NE density, digestible crude protein, digestible lysine, digestible threonine, digestible tryptophan, and digestible methionine requirements were standardized to the dietary NE (kg/MJ NE). These standardized requirements were considered as constraints to be met by diets that that will then be tailored to the pig requirements. From the calibrated nutritional profiles of the individual pigs obtained with InraPorc with the experimental data, the digestible CP and four AAs requirement per MJ NE for each individual pig were obtained from InraPorc. The individual requirement indicators were at maximum in the early stages of growth. The following requirements were averaged to obtain the representative requirement of each line *l*: digestible crude protein requirement (*Alpha*_*l*_), digestible lysine requirement (*Beta*_*l*_), digestible threonine requirement (*Gamma*_*l*_), digestible methionine requirement (*Lambda*_*l*_), and digestible tryptophan requirement (*Delta*_*l*_).

#### Nutritional objective for diet formulation

For diet formulation tailored to nutritional requirements, the linear equations 1–6 were defined as constraints for each line *l* (*l* = 2 in our study) and Q as possible ingredients (Q = 14 in our study). The first equation ensures that the prospective diet does not exceed 1 kg, and the remainders of the equations guarantee that the dietary nutrient requirements are satisfied based on the representative requirements of each line: 

$$\begin{array}{l} 1\;kg-additives\;\left(kg\right)=\overset{Q}{\underset{i=1}{\sum }}q_{il}\; \end{array}$$ (1)

$$\begin{array}{l} Alpha_{l}=\underset{i=1}{\overset{Q}{\sum }}\;q_{il}\;CP{}_{i}/\;\underset{i=1}{\overset{Q}{\sum }}\;q_{il}NE_{i\;\;\;} \end{array}$$ (2)

$$\begin{array}{l} Beta_{l}=\underset{i=1}{\overset{Q}{\sum }}\;q_{il}\;LLY_{i}/\;\underset{i=1}{\overset{Q}{\sum }}\;q_{il}NE_{i\;\;\;} \end{array}$$ (3)

$$\begin{array}{l} Gamma_{l}=\underset{i=1}{\overset{Q}{\sum }}\;q_{il}\;LTH_{i}/\underset{i=1}{\overset{Q}{\sum }}\;q_{il}NE_{i\;\;\;} \end{array}$$ (4)

$$\begin{array}{l} Delta_{l}=\underset{i=1}{\overset{Q}{\sum }}\;q_{il}\;LTR_{i}/\underset{i=1}{\overset{Q}{\sum }}\;q_{il}NE_{i\;\;\;} \end{array}$$ (5)

$$\begin{array}{l} Lambda_{l}=\underset{i=1}{\overset{Q}{\sum }}\;q_{il}\;DLM_{i}/\;\underset{i=1}{\overset{Q}{\sum }}\;q_{il}NE_{i\;\;\;} \end{array}$$ (6)

where $\;\;\;q_{il}$ (kg) is the rate of incorporation of the ith ingredient in the diet in line l, and NE_i_ (MJ), $CP_{i}$ (kg/MJ NE), $LLY_{i}(kg/MJ\;\;\;NE),\;\;\;LTH_{i}\;(kg/MJ\;\;\;NE),\;\;\;LTR_{i}\;(kg/MJ\;\;\;NE),$ and $DLM_{i}$ (kg/MJ NE) are, respectively, the net energy, digestible crude protein, digestible lysine, digestible threonine, digestible tryptophan, and digestible methionine contents of ith ingredient.

#### Line tailored diet formulation with the least cost, least environmental score, and joint cost–environment optimization objectives

In addition to covering the requirements of the genetic line selected, for each line, three optimization scenarios were considered: (1) a least-cost (**LC**) diet, (2) a diet with the least environmental impact score within an acceptable cost interval compared with the LC diet, and (3) a joint cost–environment optimized diet. First, the price normalized to the NE of the ingredient was applied to avoid formulating diets with insufficient energy content that would subsequently increase FI (Quiniou and Noblet, 2012):

$$\begin{array}{l} \;mincost=\underset{i=1}{\overset{Q}{\sum }}\;q_{il}\;\;\;p_{i}/NE_{i\;\;\;} \end{array}$$ (7)

where $\;q_{il}$, *p*_*i*_, and *NE*_*i*_ are the rate of incorporation of the *i*th ingredient in the diet targeting line *l*, the price, and net energy of *i*th ingredient, respectively, with *i* = 1,…, Q. The LC diets for each line were obtained by applying the optimization algorithm NSGA-II from the MCO library in R version 3.6.3 (with a population size of 340 and 3,500 generations) to the objective function and constraints. This algorithm identifies the non-dominated solutions on the Pareto-optimal front curve that minimize the objective function while best satisfying the constraints.

The environmental impacts (GWP_*LCl*_, AP_*LCl*_, EP_*LCl*_, and LO_*LCl*_) of the LC diet for each line *l* were calculated by summing the environmental impacts of each ingredient (Supplementary Material 6) in proportion to their rate of incorporation in the diet:

$$\begin{array}{l} impact_{LC_{l}}=\underset{i=1}{\overset{Q}{\sum }}\;q_{il}\;impact_{i} \end{array}$$ (8)

where *impact*_*i*_ is the environmental impact of ingredient *i*, and *impact* is GWP, AP, EP, or LO.

Second, the environmental objective to be minimized was computed. The environmental impacts of the LC diet of each line were used as normalization factors for each impact of the new line formulated diet (Garcia-Launay et al., 2018). Then, the impacts in an environmental impact (**EI)** score were combined linearly to obtain the objective function to minimize:

$$\begin{array}{l} EI_{score\_l}=\underset{impact=1}{\overset{4}{\sum }}\;w_{impact}((\underset{i=1}{\overset{Q}{\sum }}\;q\prime_{il}\;impact_{i}/NE_{i})/(impact_{LC_{l}}/NE_{LC_{l}})) \end{array}$$ (9)

where ${q^{\prime }}_{\mathrm{il}}$ and *NE*_*i*_ are the quantity and net energy of *i*th ingredient in the diet for line *l*, respectively. To avoid unbalanced environmental impacts of the optimized tailored diets, an equal weighting of one was used for *w*_*GWP*_, *w*_*EP*_, *w*_*AP*_, and *w*_*LO*_. The NSGA-II optimization algorithm was applied to the objective function (equation 9) to obtain the diets with the least environmental impact score under nutritional constraints (equations 1–6), plus the additional constraint that the costs of the least environmental score diets were limited to 110% of the cost of the LC diet for each line.

Third, the environmental and economic objectives were linearly integrated into one multi-objective function with normalization of each component to their counterparts for the LC diet used as a baseline, considering a weighting factor (***w***_***t***_) for EI score and its complement of 1 − *w*_*t*_ for the cost:

$$\begin{array}{ll} Joint\;\;Score_{l}= & \;w_{t}\left(\underset{impact=1}{\overset{4}{\sum }}\;w_{impact}((\underset{i=1}{\overset{Q}{\sum }}\;q{\prime \prime }_{il}\;impact_{i}/NE_{i})/(impact_{LC_{l}}/NE_{LC_{l}}))\right)\\ & +\left(1-w_{t}\right)((\underset{i=1}{\overset{Q}{\sum }}\;q{\prime \prime }_{il}\;p_{i}/NE_{i})/(\;price_{LC_{l}}/NE_{LC_{l}})) \end{array}$$ (10)

where $q{\prime \prime }_{il}$ and *NE*_*i*_ are the quantity and net energy of *i*th ingredient in the new formulated diet for line *l*, respectively.

Environmental impacts and costs were expressed relative to the net energy of the ingredients. The joint diet was obtained for each line by applying the NSGA-II optimization algorithm on the objective function (equation 10) for each *w*_*t*_ from 0 to 1 with a step of 0.01, which made it possible to investigate the impact of trade-offs between the economic and environmental objective. The best-optimized diet was when the reduction in the environmental score relative to the environmental score of the LC diet vs. the increase in price relative to the price of the LC diet became the maximum. This *w*_*t*_ point identified the optimum trade-off between the economic and environmental objectives of the formulation of feed for each line.

### Assessment of profit sensitivity of each line to market price volatility

The profit sensitivity of each line with each diet was evaluated as the percentage change in market prices that would reduce the profit of the line concerned to zero. Since the market price of pig is the only source of revenue in this study and we were focusing on feed efficiency during fattening, the sensitivities of the line were assessed only relative to an increase in the cost of the fattening diets or to a decrease in pig price. Analyzing the sensitivity of the ingredients to price volatility would require re-simulating the responses of individual pigs to the new optimized diets due to the changes in the price of the ingredients. Changing the price of an ingredient one at a time could lead to an unpredictable outcome due to the relative prices, CP, and AA content of each ingredient, while the characteristics of each ingredient are beyond the scope of this study.

### Statistical analyses

The performance traits for each pig were simulated with InraPorc in response to the reference and the optimized diets in each line and then used as input parameters for the individual trait-based bio-economic and LCA models to assess the economic and environmental impacts of the combined genetics and nutrition optimization scenarios. Statistical analyses were performed for the individual profit, environmental impacts, and performance traits. The line average (SD) of the growth performance traits and their corresponding profits and environmental impacts were computed per line, and Student’s *t*-tests were used to test the differences in all variables between the two lines (differences were considered significant at *P* < 0.05). The correlations between profit, environmental impacts, and performance traits were calculated together with their 95% confidence intervals using the cor.test function in R.

## Results

### Characteristics of optimized diets

Genetic differences were found between the requirements representative of the lines. The average (SD) requirements for digestible crude protein, digestible lysine, digestible threonine, digestible methionine, and digestible tryptophan were greater for LRFI pigs [11.75 (2.46), 0.91 (0.20), 0.58 (0.12), 0.27 (0.03), and 0.16 (0.06) g/MJ NE, respectively] compared with HRFI pigs [11.04 (2.33), 0.86 (0.18), 0.55(0.11), 0.26 (0.05), and 0.15 (0.03) g/MJ NE, respectively]. The diets with the LC and with the least environmental scores tailored to the representative requirements of each line were obtained by minimizing the corresponding objective functions. The joint optimized diet for each line was obtained from an optimum trade-off between LC and least environmental score objectives using a weighting factor of w_t_. The joint diets were obtained for *w*_*t*_ = 0.24 for LRFI and *w*_*t*_ = 0.44 for HRFI, at the point where the decrease in the environmental score (standardized to the score of the LC diet) relative to the increase in price (standardized to the price of the LC diet) was the highest. The composition of the optimized diets is provided in Table 1. The resulting environmental impacts, score, and price of 1 MJ NE of the optimized and reference diets are provided in Table 2. Expressing the environmental impacts, score, and price per MJ NE of the diet made them comparable within and between lines. In both lines, all the optimized diets had lower prices and lower environmental scores than the reference diet, with the exception of the environmental score of the LC diet in LRFI (0.430 vs. 0.416) due to greater GWP and EP. The joint diet in both lines had a greater environmental score than the least score diet of the line (0.394 vs. 0.392 for LRFI and 0.395 vs. 0.393 for HRFI) and a greater price than the LC diet of the line (0.0210 vs. 0.0201 for LRFI and 0.0206 vs. 0.0203 for HRFI). In all the optimized diets, EP increased compared with the reference diet. Finally, no systematic difference in the environmental impacts or prices was found between diets formulated for the LRFI and the HRFI pigs.

**Table 1.** Diet compositions of the reference, LC, least environmental score, and joint cost–environment optimized diets of the LRFI and HRFI lines

Ingredients	Reference	LRFI LC	HRFI LC	LRFI Least score	HRFI Least score	LRFI Joint	HRFI Joint
Net energy, MJ/kg	9.70	9.27	10.01	9.38	9.75	9.69	9.66
Oat	0	0.0	0.0	0.0	0.0	19.8	0.4
Triticale	0	545.0	170.0	53.4	1.3	217.1	157.8
Corn	0	7.0	501.0	319.0	316.0	379.3	169.7
Pea	0	28.5	38.4	0.0	160.0	47.3	88.7
Rapeseed meal	0	34.6	1.3	155.6	82.0	52.0	12.5
Sunflower meal	0	80.1	23.9	0.5	0.0	60.3	40.5
Barley	409.4	264.0	153.1	354.0	347.0	121.0	361.5
Wheat	327	1.1	2.1	74.0	44.0	33.7	107.0
Soybean meal 48	202	0	66.9	3.9	0.0	25.6	16.6
Sunflower oil	23	0	0	0	9.6	3.4	4.6
l-Lysine HCL	3.5	5.5	7.7	5.6	4.5	5.6	5.1
l-Threonine	1.4	2.0	1.9	1.7	1.8	1.9	1.9
l-Tryptophan	0.3	0.2	0.5	0.4	0.5	0.5	0.4
dl-Methionine	0.9	0.6	0.7	0.5	0.8	0.6	0.7
Salt	4.5	4.5	4.5	4.5	4.5	4.5	4.5
Calcium carbonate	11	11	11	11	11	11	11
Dicalcium phosphate	12	12	12	12	12	12	12
Vitamins and minerals	5	5	5	5	5	5	5

**Table 2.** Environmental impacts, environmental impact score, and price per unit of net energy (/MJ NE) of the reference, LC, least score, and joint cost–environment optimized diets for the LRFI and HRFI lines

/MJ NE	GWP, g CO_2_ eq	AP, g SO_2_ eq	EP, g P eq	LO, m^2^a crop eq	Environmental impact score	Price, €
Reference diet	509	0.686	0.0422	0.186	0.416	0.0241
LRFI diets						
LC	541	0.613	0.0526	0.181	0.430	0.0201
Least score	486	0.707	0.0458	0.135	0.392	0.0212
Joint	486	0.663	0.0505	0.152	0.394	0.0210
HRFI diets						
LC	483	0.683	0.0599	0.141	0.399	0.0203
Least score	442	0.648	0.0593	0.151	0.393	0.0213
Joint	490	0.643	0.0496	0.163	0.395	0.0206

### Simulated individual trait responses to the diets

The average (SD) of the performance traits predicted responses to the line-optimized diets simulated with InraPorc up to 120-kg BW is listed in Table 3. With the same reference diet, the LRFI line had lower predicted ADFI, total FI, FCR, RFI, energy conversion ratio, lipid weight, and BFT at slaughter, a longer fattening period, increased protein weight, LMP, and protein/lipid ratio at slaughter (*P* < 0.05). The ADG, BW, and carcass weight at slaughter and protein deposition (**PD**) during growth did not differ between lines (*P* > 0.14). With the optimized diets, almost the same differences were obtained, except for FCR and FI traits when expressed in kilogram of feed due to the differences in NE/kg of optimized diets between the lines. However, expressing conversion ratio in MJ (energy conversion ratio) returned the original differences. An increase in the duration of the fattening period was observed when pigs’ performances were predicted from the optimized diets compared with the reference diet. For ADG and duration of fattening, the differences between the lines increased slightly with the optimized diets, especially with the joint diet.

**Table 3.** Average (SD) and *P-*values of differences between the lines in growth performance and body composition traits^1^ in the LRFI and HRFI lines predicted with the reference, LC, least score, and joint optimized diets, as simulated by InraPorc

	Reference			LC			Least score			Joint		
	LRFI	HRFI	*P-*value^2^	LRFI	HRFI	*P-*value	LRFI	HRFI	*P-*value	LRFI	HRFI	*P-*value
ADG fattening, kg/d	0.80 (0.091)	0.83 (0.080)	0.14	0.77 (0.089)	0.80 (0.071)	<0.05	0.78 (0.089)	0.81 (0.072)	0.06	0.78 (0.090)	0.82 (0.074)	<0.05
ADFI fattening, kg/d	1.99 (0.20)	2.17 (0.16)	<0.0001	2.06 (0.21)	2.08 (0.15)	0.54	2.04 (0.21)	2.13 (0.16)	<0.05	1.98 (0.20)	2.16 (0.16)	<0.0001
FI fattening, kg	229 (20)	238(20)	<0.05	248(17)	235(17)	<0.001	242(18)	240(18)	0.60	234(18)	240 (19)	0.09
FCR fattening, kg /kg gain	2.48 (0.21)	2.62 (0.21)	<0.001	2.68 (0.17)	2.58 (0.17)	<0.01	2.61 (0.19)	2.64 (0.18)	0.55	2.53 (0.18)	2.64 (0.19)	<0.01
ECR fattening, MJ /kg gain	24.08 (2.06)	25.46 (2.06)	<0.001	24.97 (1.66)	25.96 (1.72)	<0.01	24.56 (1.81)	25.84 (1.77)	<0.001	24.59 (1.79)	25.60 (1.89)	<0.01
Fattening duration, d	116(15)	110(12)	<0.05	122(16)	114(11)	<0.01	1120 (16)	113(11)	<0.05	1120 (16)	112(11)	<0.01
BW at slaughter, kg	121 (0.4)	121 (0.5)	0.67	121 (0.4)	121(0.4)	0.88	121(0.4)	121 (0.4)	0.34	121 (0.4)	121 (0.4)	0.93
PD fattening, g/d	133 (14)	133 (13)	0.97	125(13)	127(11)	0.27	128(14)	128 (11)	0.76	128(13)	130 (11)	0.25
Carcass weight, kg	95.9 (0.33)	95.9 (0.35)	0.76	95.9 (0.33)	95.9 (0.34)	0.77	96.0 (0.35)	95.9 (0.33)	0.16	95.9 (0.31)	95.9 (0.32)	0.94
Lipid weight at slaughter, kg	23.63 (3.37)	26.99 (2.86)	<0.0001	25.27 (2.87)	28.27 (2.58)	<0.0001	24.74 (3.09)	28.08 (2.65)	<0.0001	24.77 (3.06)	27.71 (2.78)	<0.0001
BFT slaughter, mm	15.82 (1.26)	17.08 (1.07)	<0.0001	16.43 (1.07)	17.56 (0.96)	<0.0001	16.24 (1.15)	17.49 (0.99)	<0.0001	16.25 (1.14)	17.35 (1.04)	<0.0001
Protein weight at slaughter, kg	19.51 (0.48)	19.05 (0.41)	<0.0001	19.29 (0.40)	18.86 (0.37)	<0.0001	19.38 (0.44)	18.89 (0.37)	<0.0001	19.35 (0.44)	18.94 (0.39)	<0.0001
LMP, %	60.7 (2.19)	58.5 (1.86)	<0.0001	59.6 (1.86)	57.7 (1.68)	<0.0001	60.0 (2.01)	57.8 (1.72)	<0.0001	60.0 (1.99)	58.0 (1.81)	<0.0001
BP/BL at slaughter	0.84 (0.14)	0.71 (0.09)	<0.0001	0.77 (0.10)	0.67 (0.07)	<0.0001	0.79 (0.11)	0.67 (0.07)	<0.0001	0.79 (0.11)	0.69 (0.08)	<0.0001

^1^ECR, energy conversion ratio; BP/BL, ratio of body protein weight/body lipid weight at slaughter; BP, body protein content; BL, body lipid content.

^2^ *P-*values were calculated via a *t*-test of the line effect.

### Environmental assessment of the lines with the optimized diets

When the two lines were simulated with the reference diet and their tailored optimized diets, an individual LCA was performed in SimaPro based on the individual performances simulated with InraPorc to assess the environmental impacts of producing 1 kg of live pig. The resulting average (SD) of the impact categories in the two lines predicted with the different diets is summarized in Table 4. Significant differences between the lines were found in the impact categories of GWP, AP, EP, and LO in all diets (*P* < 0.05). For each optimization objective, the LRFI line, in all impact categories, had systematically smaller environmental burdens than the HRFI line using the four diet scenarios (*P* < 0.05): reference (7.21%), LC (8.11%), least score (4.91%), and joint optimized (4.29%) diets. The lines impacts predicted with the reference diet showed a maximum difference in AP and a minimum difference in LO (*P* < 0.0001). The lines with the optimized diets were predicted to systematically have lower impacts than the reference diet, except for LO for LRFI fed the LC diet and EP for all optimized scenarios. In the HRFI line, among the diets optimized for LC, least score, and joint environment and economic objectives, the maximum and minimum decreases in environmental impacts compared with the reference diet were predicted in LO (−13.21%) and GWP (−5.52%) for the LC diet. Likewise, in the LRFI line, the maximum and minimum decreases were observed in LO (−17.85%) for least score diet and in GWP (−2.54%) for the LC diet. To compute a synthetic environmental score at the farm gate similar to the environmental score defined for the diet optimization procedure, an environmental score was set up. It was defined as the sum of the four environmental impacts predicted with the considered diet divided by the sum of the environmental impacts predicted with the same line LC diet to allow comparisons across scenarios. In this way, the global environmental indicators in the LRFI line were observed in almost the same order as the order of the environmental scores of the diets (Supplementary Material 7).

**Table 4.** Average (SD) of four environmental impact categories calculated per kilogram of pig with BW of 120 kg at the farm gate through individual LCA using the ReCiPe 2016 Midpoint (H) V1.13 method and mean (SD) of profit per pig (120 kg) at farm gate resulting from the bio-economic model for the LRFI and HRFI lines predicted with the reference diet and their LC, least environmental score, and joint cost–environment optimized diets

		Reference			LC			Least score			Joint		
Impact category	Unit	LRFI	HRFI	*P-*value^1^	LRFI	HRFI	*P-*value	LRFI	HRFI	*P-*value	LRFI	HRFI	*P-*value
GWP	kg CO_2_ eq	2.07 (0.12)	2.21 (0.12)	<0.0001	2.02 (0.095)	2.09 (0.096)	<0.0001	1.96 (0.098)	2.00 (0.092)	<0.05	1.96 (0.096)	2.02 (0.098)	<0.0001
AP	g SO_2_ eq	36.8 (2.78)	40.0 (2.79)	<0.0001	33.07 (1.99)	37.1 (2.22)	<0.0001	35.6 (2.37)	36.5 (2.22)	<0.05	34.6(2.26)	35.3 (2.23)	<0.0001
EP	g P eq	1.16 (0.077)	1.24 (0.077)	<0.0001	1.39 (0.079)	1.56 (0.092)	<0.0001	1.27 (0.077)	1.39 (0.081)	<0.0001	1.36 (0.083)	1.40 (0.089)	<0.05
LO	m^2^a crop eq	4.30 (0.30)	4.58 (0.30)	<0.0001	4.35 (0.25)	3.97 (0.22)	<0.0001	3.53 (0.21)	4.17 (0.24)	<0.0001	3.89(0.23)	4.22 (0.25)	<0.0001
Profit	€/pig	11.10 (5.83)	8.50 (6.82)	<0.05	17.75 (5.56)	14.47 (7.01)	<0.01	16.28 (5.75)	12.73 (7.32)	<0.01	16.86 (5.68)	15.58 (5.64)	0.22

^1^ *P-*values were calculated via a *t*-test of the line effect.

### Individual profit per line with the optimized diets

The individual traits simulated by InraPorc for pigs predicted with their own line diet were imported into the bio-economic model to calculate the line profit for each feeding scenario. The average (SD) of the profits is given in Table 4. The difference in profits between the two lines (*P* < 0.05) and the reference diet revealed that the profit of the LRFI line was greater than that of the HRFI line. The diets that cost least and had the least score also produced greater profits in LRFI pigs (*P* < 0.01), whereas, for the joint diet, the difference between the lines was not significant (*P* > 0.22). The maximum profit in the LRFI line was predicted with the LC diet (17.75 €/pig), whereas it was obtained with the joint optimized diet in the HRFI line (15.58 €/pig).

### Correlations between individual growth performance traits and profit

To illustrate the relationships between growth performance traits and profit, phenotypic correlations were computed between the performances of individual pigs and the individual profit in each line predicted with the different diets. As the correlations were very similar for all diets in a given line, only correlations estimated with the lines outputs predicted with their own joint optimized diet are reported in Table 5. The correlations for the other diets, conventional, LC, and least score are reported in Supplementary Material 8. Profits with all optimization objectives were highly correlated with FCR (correlation < −0.82) in both lines. With ADG, the correlations were positive and moderate to high and did not differ from zero with ADFI in either line. For traits related to body and carcass composition (BFT), body protein (**BP**) content, body lipid (**BL**) content, ratio of body protein weight/body lipid (**BP/BL**) weight at slaughter, and LMP, correlations with profit were greater in the HRFI line (absolute values > 0.71) than in the LRFI line (absolute values > 0.31), with non-recovering 95% confidence intervals. The profit was highly positively correlated with PD in both lines (> +0.61). In addition to gain insights into the relationships between the environmental impacts and profits of the lines, phenotypic correlations were computed between the profits and the individual LCA results in each line. No evidence for differences between lines was found for these correlations, which were high and negative (< −0.88).

**Table 5.** Phenotypic correlations (95% confidence interval) between performance traits, environmental impacts, and profit obtained from the sale of a pig weighing 120 kg at the farm gate, with the simulated performance traits in the LRFI and HRFI lines with their joint cost–environment optimized diets

	LRFI Joint	HRFI Joint
Trait^1^		
ADG	0.57 (0.37; 0.72)	0.42 (0.18; 0.61)
FCR	−0.90 (−0.94; −0.84)	−0.85 (−0.91; −0.76)
Fattening duration	−0.58 (−0.73; −0.38)	−0.56 (−0.71; −0.35)
ADFI	0.07 (−0.19; 0.32)	−0.20 (−0.44; 0.07)
BP/BL	0.39 (0.15; 0.59)	0.75 (0.61; 0.85)
BFT	−0.47 (−0.65; −0.24)	−0.80 (−0.88; −0.68)
PD	0.73 (0.59; 0.83)	0.63 (0.45; 0.77)
BL	−0.47 (−0.65; −0.24)	−0.80 (−0.88; −0.68)
BP	0.56 (0.35; 0.71)	0.83 (0.73; 0.90)
LMP	0.48 (0.26; 0.66)	0.81 (0.69; 0.88)
Environmental impacts		
GWP	−0.90 (−0.94; −0.84)	−0.92 (−0.95; −0.86)
AP	−0.90 (−0.94; −0.84)	−0.92 (−0.95; −0.87)
EP	−0.90 (−0.94; −0.84)	−0.92 (−0.95; −0.86)
LO	−0.90 (−0.94; −0.84)	−0.92 (−0.95; −0.86)

^1^BP/BL, ratio of body protein weight/body lipid weight at slaughter; BL, body lipid content; BP, body protein content.

### Revenue and production cost breakdown

The bio-economic model made it possible to access a nonconstant cost breakdown and the revenue for each individual pig in the two lines. The average (SD) of these costs and revenue for each line and each diet is presented in Supplementary Material 9, and their costs of diet, energy, water, and labor during fattening and the profit per pig weighing 120 kg at the farm gate predicted with the reference, LC, least score, and joint optimized diets are presented in Table 6. The cost of the fattening diet within each line was significantly lower with the optimized diets than with the reference diet (*P* < 0.0001), and the decreases ranged from 10% (least score diets) to 14% for the joint diet in the LRFI line and for the joint and LC diets in the HRFI line. Significant line differences in costs, energy, water, and labor during the fattening period were observed with the reference, LC, and least score diets (*P* < 0.05). There was no line difference in the cost of the fattening diet with the joint diets (*P =* 0.74) and the cost of water with the least score diets (*P* = 0.63).

**Table 6.** Average (SD) and *P*-values of costs of diet, energy, water, and labor during fattening and the profit per pig weighing 120 kg at the farm gate in the LRFI and HRFI lines predicted with the reference, LC, least score, and joint optimized diets

	Reference			LC			Least score			Joint		
	LRFI	HRFI	*P-*value^1^	LRFI	HRFI	*P-*value	LRFI	HRFI	*P-*value	LRFI	HRFI	*P-*value
Fattening diet, €	53.7 (4.74)	55.9 (4.76)	<0.05	46.4 (3.30)	48.0 (3.56)	<0.05	48.2 (3.72)	50.0 (3.80)	<0.05	47.6 (3.65)	47.8 (3.82)	0.70
Energy, €	3.7 (0.04)	3.6 (0.04)	<0.05	3.7 (0.04)	3.6 (0.04)	<0.01	3.7 (0.04)	3.6 (0.03)	<0.01	3.7 (0.04)	3.6 (0.04)	<0.01
Water, €	2.5 (0.19)	2.5 (0.18)	<0.05	2.6 (0.17)	2.5 (0.16)	<0.001	2.6 (0.17)	2.6 (0.16)	0.60	2.5 (0.17)	2.6 (0.17)	<0.05
Fattening labor, €	4.2 (0.57)	4.0 (0.44)	<0.05	4.4 (0.60)	4.1 (0.42)	<0.01	4.3 (0.59)	4.1 (0.42)	<0.05	4.3 (0.60)	4.0 (0.42)	<0.01
Profit, €	11.1 (5.83)	8.5 (6.82)	<0.05	17.8 (5.56)	14.5 (7.01)	<0.01	16.3 (5.75)	12.7 (7.32)	<0.01	16.9 (5.68)	15.6 (5.64)	0.20

^1^ *P-*values were calculated via a *t*-test of the line effect.

### Assessment of profit sensitivity to market price volatility

**Figure 1.**
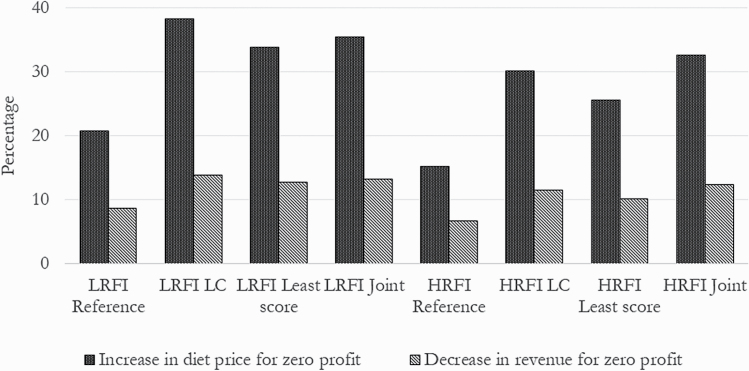
Increase percentage in the price of fattening diet and percentage reduction in the market price of a pig in each line with the reference, least score, LC, and joint cost–environment optimized diets that would result in zero profit for each line.

Figure 1 shows changes in the costs of the fattening diet and the market price of pigs that would be needed to make zero profit. In the case of an increase in the price of the diet, the HRFI line with the reference diet revealed the minimum possible changes (15.2% increase to reach zero profit), and the LRFI line with LC diet revealed the maximum possible changes (38.2%) to the increase in the price of this diet. If the price of pig were to go down, the same scenarios show a minimum margin (6.6%) and a maximum margin (13.8%), respectively. With the joint diets, the percentages in the LRFI line were close to those in the LC diet, whereas the HRFI line had the highest percentages (32.6% increase in the price of the diet and 12.2% drop in the pig market price).

## Discussion

In this study, we used individual trait-based bio-economic and LCA models to investigate the possible improvement in pig production sustainability resulting from incorporating economic and environmental impacts in diet optimization to satisfy genetically defined needs and ultimately contribute to the overall farm feed efficiency. The bio-economic model was developed specifically for this study, whereas the LCA model was previously developed, and the procedure, challenges, and limitations are reported in Soleimani and Gilbert, (2020, 2021).

### The bio-economic model

Bio-economic models are already available in the literature (e.g., de Vries, 1989; Ali et al., 2018a). The de Vries (1989) model details a sow’s life cycle. We decided not to use that model because we wanted to focus on the fattening period and consequently chose to include the costs of sows and their litters up to weaning in the cost of weaned piglets. In contrast, the fattening period is simulated in detail in our model as we decided to use the InraPorc pig growth simulator as proposed by Ali et al. (2018a) to model growth profiles. In addition, using the population version of InraPorc enabled us to simulate the growth performance traits of all individual pigs in response to the specific composition of each diet rather than the response of the average pig. Ali et al. (2018b) incorporated the environmental impact in their bio-economic model by monetizing the impact of greenhouse gases using the shadow price of CO_2_. Due to the lack of universal and standardized guidelines on how to monetize the environmental impacts, in our study, we alternated separate economic and environmental assessments of the four main categories affected by pig production (GWP, AP, EP, and LO) using individual models. The results obtained from the individual economic and environmental assessments such as correlations between profits, environmental impacts, and traits may be applicable for further relative weight assignment of the economic and environmental criteria or to attribute economic value to environmental impacts with the aim of combining economic and environmental assessment in a single economic assessment. From these results, any choice of the relative weight of the economic and environmental criteria, or choice of cost of impacts, can be applied to further combine assessments and compare scenarios. Finally, in a study of feed efficiency, one may wish to assess the economic impact of price volatility at the ingredients level. However, in tailored diet optimization, changes in the price of each ingredient would change the composition of all the diets, including the LC diet used as the baseline, which would change the composition of all optimized diets. The composition of each new optimized diet should thus be incorporated in InraPorc to simulate the new performance traits in response to new diet composition. Repeating all these procedures when the price of each ingredient changed is not feasible. Performing an economic assessment based on the performance traits of individual pigs and coupling it with individual LCA enabled us to investigate the correlations between performance traits, environmental impacts, and the final profit obtained with the lines.

### Economic and environmental evaluation of combined genetics and nutrition optimization scenarios

The differences in environmental impacts (Soleimani and Gilbert, 2020) and profit between the LRFI and HRFI lines using a single reference diet showed that pig selection for feed efficiency based on RFI alone is effective to systematically improve the sustainability of pig production even without combining this selection emphasis with diet optimization. The reference diet provided a baseline to compare the improvements due to combined genetics and nutrition optimization scenarios. If the reference diet not only was different but also covered all animal requirements, the reduction percentages of impacts and costs would be affected, but not the line comparisons obtained for the optimized diets, as the animal requirements profiles would be very similar. The high profit and low environmental score of the LRFI line with its own joint optimized diet demonstrated that combined genetics and nutrition optimization strategy can increase the sustainability with only small compromises with respect to each pillar. The profits of the lines, predicted with the reference diet, differed by 23%, which can be referred to their genetic difference. Therefore, any change in the difference between the old and new diets can mainly be interpreted as the impact of the new diet formulation. Accordingly, the decrease in the difference in profit between the lines from 23% with the reference diet to 8% (not significant) with the joint diets shows that the tailored diet formulation and optimization can alleviate the innate difference in profitability between populations with different genetic potential. Using this approach also reduced the differences in environmental impact in the two lines by half, thereby also alleviating part of the genetically related environmental burden. The joint diets for the lines were obtained with different weighting factors (*w*_*t*_), reflecting distinct trade-off points between economic and environmental objectives due to the differences in the nutritional requirements between the lines. Part of the advantage of having more efficient animals in terms of environmental impacts could then be offset by delivering a more “environment-friendly” diet to the less efficient animals. In the HRFI line, the joint optimized diet resulted in maximum profit rather than the LC diet, mainly because of greater revenue due to better market quality of the carcass. Finally, the improved robustness of the lines with the joint diet scenario vs. changes in the diet and in the market price of pigs demonstrated that tailored diet formulation combined with genetics is an effective way to achieve economically sustainable pig production. Considering the change of pig price in France from 2007 to 2020 (https://rnm.franceagrimer.fr/prix?PORC), the margins obtained with the worst scenario (HRFI with the reference diet) would lead to 34% of the weeks where the farmer would not cover the production costs by selling the pigs, whereas these situations of negative economic outcome would be reduced to 15% of the weeks for the best scenario (LRFI with LC diet). It should be noticed that a different pricing context would lead to different compositions of the optimized diets, and then differences in the predictions for all scenarios, but the main conclusions about the opportunities of the proposed approach would hold. How the approach would respond to different pricing contexts would require further automation of the predictions and assessment models, to run multiple scenarios in a separate study. In developing the bio-economic model, the cost of manure treatment and application from weaning to finishing was assumed to be offset by its revenue. Depending on the geographical context of the farm, manure could be a value or a burden for the farmer (Risse et al., 2006). However, due to low differences of manure quantities between lines as well as the market value of manure compared with the market value of pig, the benefit or burden of the manure is expected to have approximately the same low effect on the profit of individual pigs. Further sensitivity studies would be needed to evaluate scenarios with contrasted manure management situations.

To make the results comparable, both bio-economic and LCA models were built using individual performance traits, and all individuals were assessed using the same models. Individual economic and environmental assessments by trait-based models revealed correlations between performance traits, profit, and environmental impacts and provided more insights into the strategies to develop for a more sustainable pig production. The moderate correlations between ADG and the duration of the fattening period, and low with ADFI, translate into high correlations between profit and FCR. This might be due partly to some of the modeling constraints, and, to considering no variation in slaughter weight, which standardizes the outputs but is not realistic, pigs being usually slaughtered in batches. The high correlation between profit and fattening FCR reflects the high contribution of feed costs to the total costs to grow pigs in the fattening period. Moreover, the high correlation between the environmental impacts and fattening FCR (Soleimani and Gilbert, 2021) underlines the significance of fattening feed efficiency in the sustainability of the pig production systems, as already reported for pigs and other species with different approaches (Ali et al., 2018a; Yi et al., 2018; Besson et al., 2020). We also found high correlations between PD and profit and environmental impacts. They are certainly linked to the carcass pricing system used for the analysis, which favors lean carcasses, and to the costs of incorporating protein-rich ingredients in the diet. This shows that traits linked to PD are the right ones to incorporate in selection for more sustainable pig production. It should be noted that in the more efficient line, the correlations between leanness and profit were not as high as they were in the less efficient line. We hypothesize that this is due to less variance in these traits in the LRFI line and hence in less sensitivity of the price of more efficient animals to a payment system based on leanness. High negative correlations between environmental impacts and the profit of the lines for all diets can be interpreted as the close link between FI and environmental impacts on the one hand, and profit on the other hand, which again underlines the importance of feed efficiency in response to the economic and environmental pillars of sustainability. The optimized diets generally had low environmental scores and their cost was low compared with the reference diet, which shows a marked potential for economic and environmental improvements in diet optimization alone. The marginally greater price of the joint diets per MJ NE relative to the LC diets (within line) showed that an optimized diet (e.g., the joint diet) can be achieved with a small compromise relative to the price of the LC diet. The increase in the duration of the fattening period for pigs performance predicted with the optimized diets compared with that of pigs performance with the reference diet may be explained by the fact that a few pigs are not satisfied in the very early growth stages because the line average of the maximum requirements is considered as constraints in diet formulation. A multiphase feeding strategy or establishing the representative requirements to the 75% quantile of the maximum pig requirements per line could compensate for this reduction in growth performance, although certainly at the expense of more spillage and increased costs and impacts. It is notable that despite the increase in the duration of the fattening period, marked economic and environmental advantages were achieved with the combined genetics and nutrition optimization scenarios, which would encourage a more overall approach to evaluate production systems, where performance losses could be offset by gains in other dimensions (for instance, feeding costs and carcass quality). This would be particularly advantageous for farmers whose feeding system does not allow for much flexibility, e.g., on-farm production systems where breeding highly efficient animals requires greater concentrations of AA and CP per MJ of NE, which might not be the most efficient choice in such systems, as it would require increasing levels of high-protein ingredients imported in the farm, or delivering unbalanced diets to highly efficient pigs, whose nutritional requirements would not be met and which then would fail to achieve their promised performances (Gilbert et al., 2017). Greater improvement in feed efficiency would be expected from individual tailored formulations compared with line tailored formulations. The variability in the input parameters such as the price of ingredients, their availability, and the environmental impacts of their production could be dynamically imported into the optimization algorithm, and tailored diet formulation and real-time optimization would not lag far behind expectations. In addition, selection indexes could be improved by incorporating traits that are highly correlated with new objectives, such as environment. The results of this study are limited to the simulation tools and choices applied, which are potentially subjected to deviation from predictions under field conditions. Therefore, further field studies will be required to confirm these predictions.

### Consistency in the implementation of combined genetics and nutrition optimization

In the present study, consistency in combined genetics and nutrition optimization processes was obtained by considering NE as the core linkage between genetics, diet formulation, and optimization. Extraction of individual requirements standardized to NE as well as standardized prices and environmental impacts of the dietary ingredients to NE provides consistency in the whole process of the combined genetic and nutrient optimization. The incorporation of standardized individual requirements to NE among the constraints of diet formulation will make it possible to control the excretion of nutrients that originates from unbalanced dietary nutrients. Mackenzie et al. (2016) included a module to estimate nitrogen excretion at the farm level in their diet formulation process, whereas, in our approach, due to the uniformity of the nutrient composition of the diets relative to NE ratios, the same excretion would be expected with all diets without the need for estimation. The incorporation of standardized prices and the impacts of ingredients in the objective functions ranked the ingredients according to their economic and environmental cost per MJ NE, which, in turn, optimized their relative rate of incorporation according to their value relative to MJ NE. One important advantage, consistency with NE throughout the process, makes it possible to predict and qualitatively compare the final farm profit and environmental impacts using the standardized price and the environmental score of the diets.

## Conclusions

Improving feed efficiency in pigs can be achieved by improving animal genetics and the composition of their diet. Genetic selection to improve feed efficiency has systematically improved the sustainability of pig production in terms of profitability and environmental impacts. Tailored diet optimization was shown to effectively improve environmental impacts and farm profitability, by minimizing the difference between nutritional requirements and supply while simultaneously orienting dietary improvement toward intended single- or multi-objective optimization of the production system. Combining genetic selection for feed efficiency and tailored diet optimization is a promising way to make pig production more sustainable and more efficient. The normalization to NE of animal nutritional requirements, diet prices, environmental impacts, and nutritional characteristics of ingredients provides consistency in the whole optimization procedure and could be considered in further precision farming developments.

## Supplementary Material

skab051_suppl_Supplementary_Materials

## Abbreviations

ADFI: average daily feed intake
AP: terrestrial acidification potential
BFT: back fat thickness
BW: body weight
EI: environmental impact
EP: freshwater eutrophication potential
FCR: feed conversion ratio
GWP: global warming potential
HRFI: high residual feed intake
LC: least cost
LCA: life cycle assessment
LMP: lean meat percentage
LO: land occupation
LRFI: low residual feed intake
LW: live weight
PD: protein deposition
RFI: residual feed intake
w_t_: weighting factor
