# Supplementary material for: Economic and environmental assessments of combined genetics and nutrition optimization strategies to improve the efficiency of sustainable pork production
Source: J Anim Sci. 2021 Feb 15;99(3):skab051. doi: 10.1093/jas/skab051 (PMC7999619; doi:10.1093/jas/skab051)
Supplement: skab051_suppl_Supplementary_Materials [file skab051_suppl_supplementary_materials.docx]

**Supplementary material 1.** Scheme of the procedure implemented for economic and environmental assessment of overall farm feed efficiency strategies.

Experimental data for two genetic lines, reference diet

Trait- based LCA model

**Environmental assessment**

Trait- based bio-economic model

**Economic** **assessment**

Statistical comparisons

of line & diet scenarios

*Individual impacts*

*Individual profit*

Simulation of individual responses to the optimised diets

**InraPorc^®^**

Set up of individual growth profiles

**InraPorc**^®^

**Estimation of individual requirements**

α = CP /MJ NE, β = Lysine/MJ NE, **γ** = Threonine/MJ NE, δ = Tryptophane/MJ NE, λ = Methionine/MJ NE

**Line requirements indicators**

α _line_ =Mean α_max 1..57_ ;β_line_ =Mean β_max1...57_; **γ** _line_ **=**Mean **γ**_max1...57_; δ _line_ =Mean δ_max1…57_; λ _line_ =Mean λ_max1…57_

*Cost & environment*

Objective

α, β, γ, δ, λ

Constraints

**Diet optimisation**

*Individual ADG, ADFI, LMP, Duration, BFT*

*Individual ADG, ADFI, LMP, dressing percentage, Fattening duration, weaning age and weight*

**Supplementary material 2.** Market price of items applied in bio-economic model and diet optimisation.

| **Item** | **Price**  **(€ per unit)** | **Reference** |
| --- | --- | --- |
| Barley (France) | 0.167 €/kg | Note de conjoncture Aliment April 2020 (IFIP) |
| Wheat soft (France) | 0.18 €/kg | Note de conjoncture Aliment April 2020 (IFIP) |
| Soybean meal 48 (South America) | 0.348 €/kg | Note de conjoncture Aliment April 2020 (IFIP) |
| Sunflower oil (France) | 0.705 €/kg | Note de conjoncture Aliment April 2020 (IFIP) |
| Corn (France) | 0.178 €/kg | Note de conjoncture Aliment April 2020 (IFIP) |
| Oat (France) | 0.192 €/kg | Note de conjoncture Aliment April 2020 (IFIP) |
| Pea (France) | 0.231 €/kg | Note de conjoncture Aliment April 2020 (IFIP) |
| Triticale (France) | 0.158 €/kg | Note de conjoncture Aliment April 2020 (IFIP) |
| rapeseed meal (France) | 0.252 €/kg | Note de conjoncture Aliment April 2020 (IFIP) |
| sunflower meal (France) | 0.182 €/kg | Note de conjoncture Aliment April 2020 (IFIP) |
| L-Lysine HCL (France) | 1.175 €/kg | Note de conjoncture Aliment April 2020 (IFIP) |
| L-Threonine (France) | 1.1 €/kg | Note de conjoncture Aliment April 2020 (IFIP) |
| L-Tryptophan (France) | 6.5 €/kg | Note de conjoncture Aliment April 2020 (IFIP) |
| DL-Methionine (France) | 1.9 €/kg | Note de conjoncture Aliment April 2020 (IFIP) |
| Salt (France)  (Sodium Chloride) | 0.112 €/kg | Note de conjoncture Aliment April 2020 (IFIP) |
| Calcium carbonate (France) | 0.05 €/kg | Note de conjoncture Aliment April 2020 (IFIP) |
| Dicalcium phosphate (France) | 0.51 €/kg | Note de conjoncture Aliment April 2020 (IFIP) |
| Oligo Vitamin (France) | 1€/kg | Note de conjoncture Aliment April 2020 (IFIP) |
| Post weaning 7kg | 35.28 € | [https://rnm.franceagrimer.fr/prix?PORCELET#](https://rnm.franceagrimer.fr/prix?PORCELET) |
| Water | 3.57 €/m^3^ | https://www.ledauphine.com/france-monde/2017/12/21/eau-quel-est-vraiment-le-juste-prix |
| Electricity | 0.0771 €/kWh | <https://www.kelwatt.fr/guide/prix-electricite-france> |
| Labor cost | 0.036 €/pig/day | Calculated based on the IFIP information (2.3 workers/200 sows) and SMIC = 10.03 €/hour |
| 100kg carcass & LMP 56% | 129.30 €/carcass | https://rnm.franceagrimer.fr/prix?PORC |
| Buildings and capital costs | 0.03 €/pig/day | It is calculated based on total investment per sow: 4937 €/sow/place; Les bâtiments en France; Les coûts pour 3 types d’élevages. IFIP report- 2019. |
| Investment | 4937 €/sow/place | Les bâtiments en France ; Les coûts pour 3 types d’élevages. IFIP report- 2019. |
| Health cost | 0.89 €/ pig | Description, evaluation, and validation of the Teagasc Pig Production Model. Calderón 2019 |
| Insurance | 1.04 €/ pig | Description, evaluation, and validation of the Teagasc Pig Production Model. Calderón 2019 |
| Maintenance & Repairs | 1.04 €/ pig | Description, evaluation, and validation of the Teagasc Pig Production Model. Calderón 2019 |
| Starter (1^st^ age)  weaning (2^nd^ age feed) | 350€/T  320€/T | <https://www.ifip.asso.fr/PagesStatics/resultat/partenaire/tele/criteres%20GTE.pdf>  page 46. |

**Supplementary material 3.** Correction factor for quantity and quality deviations from the baseline price for a carcass weight of 100kg and lean meat percentage of 56%.

https://www.gis-elevages-demain.org/content/download/3429/34955/version/1/file/m%C3%A9moire_ElodieLopez_rectoverso.pdf

| PORKS | | | WEIGHT RANGE | | | | | | | | | | | | |
| --- | --- | --- | --- | --- | --- | --- | --- | --- | --- | --- | --- | --- | --- | --- | --- |
| LMP | Deviation | Total deviation | 45  69.9 | 70  77.9 | 78  79.9 | 80  81.9 | 82  86.9 | 87  99 | 99.1  105 | 105.1  106 | 106.1  107 | 107.1  108 | 108.1  109 | 109.1  110 | 110.1  120 |
|  |  |  | -0.30 | -0.18 | -0.10 | -0.02 | 0.00 | 0.02 | 0.00 | -0.04 | -0.10 | -0.12 | -0.14 | -0.16 | -0.20 |
| >=64 | -0.01 | 0.16 | -0.14 | -0.02 | 0.06 | 0.14 | 0.16 | 0.18 | 0.16 | 0.12 | 0.06 | 0.04 | 0.02 | 0.00 | -0.04 |
| 63 | 0.00 | 0.17 | -0.13 | -0.01 | 0.07 | 0.15 | 0.17 | 0.19 | 0.17 | 0.13 | 0.07 | 0.05 | 0.03 | 0.01 | -0.03 |
| 62 | 0.00 | 0.17 | -0.13 | -0.01 | 0.07 | 0.15 | 0.17 | 0.19 | 0.17 | 0.13 | 0.07 | 0.05 | 0.03 | 0.01 | -0.03 |
| 61 | 0.02 | 0.17 | -0.13 | -0.01 | 0.07 | 0.15 | 0.17 | 0.19 | 0.17 | 0.13 | 0.07 | 0.05 | 0.03 | 0.01 | -0.03 |
| 60 | 0.03 | 0.15 | -0.15 | -0.03 | 0.05 | 0.13 | 0.15 | 0.17 | 0.15 | 0.11 | 0.05 | 0.03 | 0.01 | -0.01 | -0.05 |
| 59 | 0.04 | 0.12 | -0.18 | -0.06 | 0.02 | 0.10 | 0.12 | 0.14 | 0.12 | 0.08 | 0.02 | 0.00 | -0.02 | -0.04 | -0.08 |
| 58 | 0.04 | 0.08 | -0.22 | -0.10 | -0.02 | 0.06 | 0.08 | 0.10 | 0.08 | 0.04 | -0.02 | -0.04 | -0.06 | -0.08 | -0.12 |
| 57 | 0.04 | 0.04 | -0.26 | -0.14 | -0.06 | 0.02 | 0.04 | 0.06 | 0.04 | 0.00 | -0.06 | -0.08 | -0.10 | -0.12 | -0.16 |
| 56 | 0.00 | 0.00 | -0.30 | -0.18 | -0.10 | -0.02 | 0.00 | 0.02 | 0.00 | -0.04 | -0.10 | -0.12 | -0.14 | -0.16 | -0.20 |
| 55 | -0.02 | -0.02 | -0.32 | -0.20 | -0.12 | -0.04 | -0.02 | 0.00 | -0.02 | -0.06 | -0.12 | -0.14 | -0.16 | -0.18 | -0.22 |
| 54 | -0.02 | -0.04 | -0.34 | -0.22 | -0.14 | -0.06 | -0.04 | -0.02 | -0.04 | -0.08 | -0.14 | -0.16 | -0.18 | -0.20 | -0.24 |
| 53 | -0.04 | -0.08 | -0.38 | -0.26 | -0.18 | -0.10 | -0.08 | -0.06 | -0.08 | -0.12 | -0.18 | -0.20 | -0.22 | -0.24 | -0.28 |
| 52 | -0.04 | -0.12 | -0.42 | -0.30 | -0.22 | -0.14 | -0.12 | -0.10 | -0.12 | -0.16 | -0.22 | -0.24 | -0.26 | -0.28 | -0.32 |
| 51 | -0.08 | -0.20 | -0.50 | -0.38 | -0.30 | -0.22 | -0.20 | -0.18 | -0.20 | -0.24 | -0.30 | -0.32 | -0.34 | -0.36 | -0.40 |
| <=50 | -0.20 | -0.40 | -0.70 | -0.58 | -0.50 | -0.42 | -0.40 | -0.38 | -0.40 | -0.44 | -0.50 | -0.52 | -0.54 | -0.56 | -0.60 |

LMP = lean meat percentage

**Supplementary material 4.** The following formulations have been applied to calculate the individual profit.

| **Cost** | **Formulation** |
| --- | --- |
| Fattening_Diet_Cost | ADFI_Fattening * Fattening_Duration* Fattening_Diet_Price |
| Postweaning_Diet_Cost | Starter_Duration * ADFI_Starter * StarterDiet_Price  + Weaning_Diet_Price * (Weaning_Duration_6_19 * ADFI_weaning_6_19 + Weaning_19_InitialFattening_Duration * ADFI_weaning_19_InitialFattening) |
| Total_Diet_Cost | Fattening_Diet_Cost + Postweaning_Diet_Cost |
| Energy_Cost | Energy_Consumption * Energy_Price * (BW_End_Fattening - BW_Weaning) |
| Water_Cost | Water_Price * (Water_To_Feed_Fattening _ratio * ADFI_Fattening * Fattening_Duration  + Water_To_Feed_PostWeaning _ ratio * (Starter_Duration * ADFI_Starter + Weaning_Duration_6_19 * ADFI_weaning_6_19 + Weaning_19_InitialFattening_Duration * ADFI_weaning_19_InitialFattening)) |
| Fattening_Labor_Cost | Worker_Cost * Duration_Fattening  Worker cost calculation per pig per day:  2.3 workers for farm with 200 sows,  2.3/200 = 0.0115 workers/sow  Each sow produces 25 weaned piglets per year on average,  0.0115/25=0.00046 workers/pig  Flat-rate remuneration for work: SMIC/hour SMIC = 10.03 €/hour before taxes  Worker cost per day = SMIC * 8 hours/day = 80.24 €/day  Worker cost per pig per day= 80.24 €/day *0.00046 workers/pig = 0.036 €/pig/day |
| PostWeaning_Labor_Cost | Worker_Cost * PostWeaning_Duration |
| Total_Labor_Cost | Fattening_Labor_Cost + PostWeaning_Labor_Cost |
| Weaning_Cost | Weaned_Piglet_price |
| Building_and_Capital_Cost | Capital_Cost * (PostWeaning_Duration + Fattening_Duration)  Capital costs calculation per pig per day:  Total investment per sow: 4937 €/sow/place  Interest rate = 6% per year  Interest cost = 0.06(6%) * 4937 €/sow = 296.22 €/sow/year  Interest cost per pig per day = 296.22/365/25 = 0.03 €/pig/day |
| Total_Cost | Total_Diet_Cost  + Energy_Cost  + Water_Cost  + Total_Labor_Cost  + Weaning_Cost  + Building_and_Capital_Cost  + Health_Cost  + Insurance_Cost  + Maintenance_Repair_Cost |
| Market price (1 pig alive) | Market_price (1 pig alive) = Market price (full carcass)  Market price (full carcass) =  [Market reference price (100kg carcass & LMP 56%)/100  + Carcass weight price correction + LMP price correction] * Carcass_Weight  Carcass_Weight = LiveBW_farm_gate * Dressing percentage/100 |
| Revenue | Market price (1 pig alive) |
| Profit | Revenue - Total_Cost |

**Supplementary material 5.**

The following formulations have been applied to calculate the emissions and excretions using the mass-balance approach (from Soleimani and Gilbert, 2020a).

| eBW= 5.969 * BP 0.944 + 0.854 * BL 0.944 | (van Milgen et al., 2008) |
| --- | --- |
| Lean meat percentage = 72.58 – 43.49 * BL/ eBW | (van Milgen et al., 2008) |
| N Body = e^(-0.9892 – 0.0145 * Lean%) * eBW^(0.7518 + 0.0044 Lean%) / 6.25 | (Dourmad et al., 1992) |
| N Intake = Feed Intake * N Feed |  |
| N Excreted = N Intake – N Retained |  |
| P _Body_ (g) = 5.39 * eBW | (Rigolot et al., 2010a) |
| Ca _Body_ (g) = 8.56 * eBW | (Rigolot et al., 2010a) |
| K _Body_ (g) = -0.0041 * eBW^2^ + 2.68 * eBW | (Rigolot et al., 2010a) |
| Cu _Body_ (mg) = 1.1 * eBW | (Rigolot et al., 2010a) |
| Zn _Body_ (mg) = 20.6 * eBW | (Rigolot et al., 2010a) |
| N_2_0 = 0.002 * N Excreted | (Rigolot et al., 2010b) |
| N_2_ = 5 * N_2_0 | (Rigolot et al., 2010b) |
| NH_3 Building_ (kg) = 17 / 14 * 0.24 * N Excreted | (Rigolot et al., 2010b) |
| ResD = Feed Intake * Residue Feed | (Rigolot et al., 2010b) |
| ECH_4 growing_ = ResD * 670 | (Rigolot et al., 2010a) |
| CH_4_ _Emitted_ = ECH_4_ / 56.65 | (Rigolot et al., 2010a) |
| CH_4_ _Housing_ (kg) = VS * B_0_ * MCF | (Rigolot et al., 2010b) |
| OM _Faeces_ = Feed * OM_feed_ * (1 – dCOM) | (Rigolot et al., 2010a) |
| dCOM = (0.744 + (14.69 DE – 0.50 NDF – 1.54 MM) / DM) / (OM / DM) | (Rigolot et al., 2010a) |

eBW = empty body weight ; BP = body protein ; L = body lipid; N Body = nitrogen content of body; N Intake = total uptaken nitrogen; N Feed = nitrogen content of 1kg feed; N Excreted = total excreted nitrogen; NRetained = nitrogen retained in the body; OM = organic matter; MM = mineral mater; DM = dry matter; dCOM = feed organic matter digestibility coefficient; NDF = Neutral detergent fiber; B0 = maximum CH4 producing capacity; MCF = methane conversion factor; ResD = digested fibre ingested.CH4 = methane; N = nitrogen; Ca = calcium; P = phosphorus; K = potassium; Cu = copper; Zn = zinc.

**Supplementary material 6.** Digestible crude protein (CP) and amino acids, and net energy (NE) of the ingredients retained for diet formulation, and their environmental impacts.

| Ingredients | CP  (g/  kg_feed_) | Lys.  (g/  kg_feed_) | Thr.  (g/  kg_feed_) | Trp  (g/  kg_feed_) | Met  (g/  kg_feed_) | NE  (MJ/  kg) | GWP  (kg CO_2_ eq) | AP  (g  SO_2_ eq) | EP  (g  P eq) | LO  (m^2^a crop) |
| --- | --- | --- | --- | --- | --- | --- | --- | --- | --- | --- |
| Barley | 80.5 | 2.85 | 2.62 | 1.03 | 1.43 | 9.56 | 0.46 | 5.60 | 0.16 | 1.371 |
| Oat | 74.2 | 2.99 | 2.36 | 0.94 | 1.51 | 8.06 | 0.50 | 7.95 | 0.20 | 2.079 |
| Triticale | 83.4 | 3.24 | 2.71 | 1.06 | 1.53 | 10.40 | 0.48 | 5.43 | 0.19 | 1.837 |
| Corn | 69.8 | 1.92 | 2.49 | 0.40 | 1.55 | 11.20 | 0.33 | 7.11 | 0.12 | 1.033 |
| Pea | 165.8 | 12.45 | 5.93 | 1.31 | 1.60 | 9.75 | 0.37 | 3.65 | 0.57 | 2.663 |
| Rapeseed meal | 254.7 | 13.5 | 10.87 | 3.28 | 6.00 | 6.26 | 0.40 | 5.36 | 0.10 | 1.211 |
| Sunflower meal | 273.5 | 9.68 | 9.72 | 3.44 | 6.99 | 5.50 | 0.25 | 2.94 | 0.25 | 1.975 |
| Wheat soft | 92.8 | 2.51 | 2.66 | 1.14 | 1.51 | 10.54 | 0.42 | 7.96 | 0.129 | 1.330 |
| Soybean meal | 391 | 25.02 | 15.4 | 5.25 | 5.89 | 7.86 | 1.52 | 5.64 | 0.385 | 2.086 |
| Sunflower oil | 0 | 0 | 0 | 0 | 0 | 29.76 | 1.17 | 15.51 | 1.12 | 8.701 |
| L-Lysine HCL | 954 | 798 | 0 | 0 | 0 | 11.88 | 10.55 | 76.60 | 37.85 | 3.118 |
| L-Threonine | 731 | 0 | 990 | 0 | 0 | 11.11 | 10.62 | 84.23 | 37.16 | 3.109 |
| L-Tryptophan | 853 | 0 | 0 | 985 | 0 | 11.53 | 21.24 | 168.47 | 74.32 | 6.219 |
| DL-Methionine | 584 | 0 | 0 | 0 | 990 | 10.61 | 2.99 | 8.86 | 0.270 | 0.016 |

CP = crude protein; LO = land occupation; EP= eutrophication potential; AP = acidification potential; GWP= global warming potential; NE = net energy; P = phosphorous; m^2^a crop= area time; NE density and digestible CP and amino acids (lysine, threonine, tryptophan, and methionine) of the ingredients were extracted from the INRA-AFZ database of feed ingredients. The environmental impacts of diet ingredients (GWP, AP, EP, LO) were obtained from the Ecoalim dataset of the AGRIBALYSE^®^ database with the Recipe method 2016.

**Supplementary material 7.** Global environmental indicator of LRFI and HRFI lines obtained from the sum of the four impact categories with weighing of one, normalised to the corresponding of the least cost diet.

| Impact category | Unit | **LRFI** | | | | **HRFI** | | | |
| --- | --- | --- | --- | --- | --- | --- | --- | --- | --- |
|  |  | Least cost | Reference | Joint | Least score | Least cost | Reference | Joint | Least score |
| Global warming potential | kg CO_2_ eq | 2.024565039 | 2.07743 | 1.956578342 | 1.964893997 | 2.0940194 | 2.216420683 | 2.0289206 | 2.00876661 |
| Acidification | kg SO_2_ eq | 0.033078762 | 0.036778 | 0.03455181 | 0.035635412 | 0.0371808 | 0.040003775 | 0.0353864 | 0.03655861 |
| Eutrophication | kg P eq | 0.001390904 | 0.001168 | 0.001365895 | 0.00127469 | 0.0015651 | 0.001240719 | 0.0014021 | 0.00139793 |
| Land occupation | m^2^a crop eq | 4.357045629 | 4.306144 | 3.897016388 | 3.537422797 | 3.9773762 | 4.583056232 | 4.2243881 | 4.17354862 |
| Sum of four impacts | | **6.416080334** | **6.42152** | **5.889512435** | **5.539226896** | **6.1101416** | **6.840721409** | **6.2900971** | **6.22027177** |
| **Global environmental indicator** | | **1** | **1.0008** | **0.9179** | **0.8633** | **1** | **1.1195** | **1.0294** | **1.018** |

[GWP (kg) + AP (kg) + EP (kg) + LO (m^2^)] _diet_ / [GWP (kg) + AP (kg) + EP (kg)+ LO (m^2^)]_Least cost diet_

**Supplementary material 8.** Phenotypic correlations (95% confidence interval) between profit of a 120kg pig and performance traits and environmental impacts , with the recorded traits in the LRFI and HRFI lines with least cost, least environmental score, joint cost-environment diet optimizations, and the reference diet.

| Trait | LRFI  Reference | HRFI  Reference | LRFI  Least Cost | HRFI  Least Cost | LRFI  Least Score | HRFI  Least Score | LRFI  Joint | HRFI  Joint |
| --- | --- | --- | --- | --- | --- | --- | --- | --- |
| ADG | 0.67  (0.49 ; 0.79) | 0.52  (0.30 ; 0.69) | 0.77  (0.64 ; 0.86) | 0.41  (0.16 ; 0.60) | 0.58  (0.38 ; 0.73) | 0.43  (0.19 ; 0.62) | 0.57  (0.37 ; 0.72) | 0.42  (0.18 ; 0.61) |
| FCR | -0.89  (-0.93 ; -0.82) | -0.88  (-0.93 ; -0.80) | -0.82  (-0.89 ; -0.71) | -0.89  (-0.93 ; -0.82) | -0.90  (-0.94 ; -0.84) | -0.90  (-0.94 ; -0.83) | -0.90  (-0.94 ; -0.84) | -0.85  (-0.91 ; -0.76) |
| Fattening duration | -0.67  (-0.79 ; -0.50) | -0.64  (-0.77 ; -0.45) | -0.80  (-0.87 ; -0.68) | -0.56  (-0.72 ; -0.35) | -0.58  (-0.73 ; -0.38) | -0.58  (-0.73 ; -0.37) | -0.58  (-0.73 ; -0.38) | -0.56  (-0.71 ; -0.35) |
| ADFI | 0.08  (-0.18 ; 0.33) | -0.12  (-0.37 ; 0.15) | 0.26  (0.01 ; 0.49) | -0.15  (-0.40 ; 0.11) | 0.06  (-0.20 ; 0.31) | -0.15  (-0.40 ; 0.11) | 0.07  (-0.19 ; 0.32) | -0.20  (-0.44 ; 0.07) |
| BP/BL | 0.40  (0.17 ; 0.60) | 0.71  (0.56 ; 0.82) | 0.31  (0.06 ; 0.53) | 0.73  (0.58 ; 0.83) | 0.40  (0.16 ; 0.60) | 0.72  (0.57 ; 0.83) | 0.39  (0.15 ; 0.59) | 0.75  (0.61 ; 0.85) |
| BFT | -0.47  (-0.65 ; -0.25) | -0.78  (-0.87 ; -0.66) | -0.33  (-0.54 ; -0.08) | -0.79  (-0.87 ; -0.66) | -0.47  (-0.65 ; -0.25) | -0.78  (-0.87 ; -0.66) | -0.47  (-0.65 ; -0.24) | -0.80  (-0.88 ; -0.68) |
| PD | 0.81  (0.70 ; 0.89) | 0.70  (0.54 ; 0.81) | 0.88  (0.80 ; 0.93) | 0.61  (0.42 ; 0.75) | 0.74  (0.59 ; 0.84) | 0.63  (0.45 ; 0.77) | 0.73  (0.59 ; 0.83) | 0.63  (0.45 ; 0.77) |
| BL | -0.47  (-0.65 ; -0.25) | -0.78  (-0.87 ; -0.66) | -0.33  (-0.54 ; -0.08) | -0.79  (-0.87 ; -0.66) | -0.47  (-0.65 ; -0.25) | -0.78  (-0.87 ; -0.66) | -0.47  (-0.65 ; -0.24) | -0.80  (-0.88 ; -0.68) |
| BP | 0.56  (0.35 ; 0.71) | 0.83  (0.73 ; 0.90) | 0.42  (0.19 ; 0.61) | 0.84  (0.74 ; 0.90) | 0.58  (0.37 ; 0.72) | 0.84  (0.74 ; 0.90) | 0.56  (0.35 ; 0.71) | 0.83  (0.73 ; 0.90) |
| LMP | 0.49  (0.26 ; 0.66) | 0.79  (0.67 ; 0.87) | 0.34  (0.10 ; 0.55) | 0.80  (0.68 ; 0.88) | 0.49  (0.27 ; 0.66) | 0.80  (0.68 ; 0.88) | 0.48  (0.26 ; 0.66) | 0.81  (0.69 ; 0.88) |
| GWP | -0.92  (-0.95 ; -0.86) | -0.93  (-0.96 ; -0.89) | -0.88  (-0.93 ; -0.81) | -0.95  (-0.97 ; -0.91) | -0.90  (-0.94 ; -0.84) | -0.95  (-0.97 ; -0.91) | -0.90  (-0.94 ; -0.84) | -0.92  (-0.95 ; -0.86) |
| AP | -0.91  (-0.95 ; -0.86) | -0.94  (-0.96 ; -0.89) | -0.89  (-0.93 ; -0.82) | -0.95  (-0.97 ; -0.92) | -0.91  (-0.94 ; -0.85) | -0.95  (-0.97 ; -0.92) | -0.90  (-0.94 ; -0.84) | -0.92  (-0.95 ; -0.87) |
| EP | -0.92  (-0.95 ; -0.86) | -0.93  (-0.96 ; -0.89) | -0.88  (-0.93 ; -0.81) | -0.95  (-0.97 ; -0.91) | -0.90  (-0.94 ; -0.84) | -0.95  (-0.97 ; -0.91) | -0.90  (-0.94 ; -0.84) | -0.92  (-0.95 ; -0.86) |
| LO | -0.92  (-0.95 ; -0.86) | -0.93  (-0.96 ; -0.89) | -0.88  (-0.93 ; -0.81) | -0.95  (-0.97 ; -0.91) | -0.90  (-0.94 ; -0.84) | -0.95  (-0.97 ; -0.91) | -0.90  (-0.94 ; -0.84) | -0.92  (-0.95 ; -0.86) |

LO = land occupation; EP= eutrophication potential; AP = acidification potential; GWP= global warming potential; BW = body weight; ADG = average daily gain; ADFI = average daily feed intake; FCR = feed conversion ratio; ECR = energy conversion ratio; PD = protein deposition; BFT = back fat thickness; BP/BL = ratio of body protein weight/ body lipid weight at slaughter. BP = body protein content; BL = body lipid content; LMP = lean meat percentage.

**Supplementary material 9.** Average (SD) of the costs and revenue of the lines fed the reference diet, least cost diet, least score diet and joint diet.

|  |  | Fattening diet cost (€) | Energy (€) | Water (€) | Fattening Labor (€) | Total Cost (€) | Revenue |
| --- | --- | --- | --- | --- | --- | --- | --- |
| Reference | LRFI | 53.67^a^ (4.74) | 3.651^a^ (0.040) | 2.45 ^a^ (0.19) | 4.18 ^a^ (0.57) | 117.39^a^ (5.61) | 128.49 (1.83) |
|  | HRFI | 55.88^b^ (4.76) | 3.631^b^ (0.043) | 2.54 ^b^ (0.19) | 3.97 ^b^ (0.44) | 119.38^b^ (5.35) | 127.88 (2.58) |
| Least cost | LRFI | 46.40^a^ (3.30) | 3.652 ^a^ (0.040) | 2.63 ^a^ (0.16) | 4.38 ^a^ (0.60) | 110.68 (4.13) | 128.44^a^ (2.33) |
|  | HRFI | 48.03^b^ (3.56) | 3.630 ^b^ (0.039) | 2.51 ^b^ (0.15) | 4.09 ^b^ (0.42) | 111.71 (3.99) | 126.18^b^ (3.70) |
| Least score | LRFI | 48.18^a^ (3.72) | 3.654 ^a^ (0.041) | 2.57 (0.17) | 4.30 ^a^ (0.58) | 112.24 (4.56) | 128.53^a^ (2.25) |
|  | HRFI | 49.98^b^ (3.80) | 3.630 ^b^ (0.039) | 2.56 (0.16) | 4.06 ^b^ (0.42) | 113.67 (4.24) | 126.40^b^ (3.71) |
| Joint | LRFI | 47.59 (3.65) | 3.651 ^a^ (0.040) | 2.50 (0.17) | 4.30 ^a^ (0.59) | 111.58 (4.49) | 128.45^a^ (2.23) |
|  | HRFI | 47.82 (3.82) | 3.630 ^b^ (0.042) | 2.56 (0.17) | 4.01 ^b^ (0.42) | 111.42 (4.34) | 127.00^b^ (2.44) |

^a,b^ means significant line difference with the same type of diet (*P* < 0.05).
